# Leveraging Social Media to Promote Public Health Knowledge: Example of Cancer Awareness via Twitter

**DOI:** 10.2196/publichealth.5205

**Published:** 2016-04-28

**Authors:** Songhua Xu, Christopher Markson, Kaitlin L Costello, Cathleen Y Xing, Kitaw Demissie, Adana AM Llanos

**Affiliations:** ^1^ College of Computing Sciences Department of Information Systems New Jersey Institute of Technology Newark, NJ United States; ^2^ Rutgers School of Communication and Information Department of Library and Information Science New Brunswick, NJ United States; ^3^ School of Public Health Epidemiology Rutgers University Piscataway, NJ United States; ^4^ School of Public Health and Cancer Institute of New Jersey Epidemiology Rutgers University Piscataway, NJ United States

**Keywords:** awareness, breast cancer, colorectal cancer, disparities, lung cancer, prostate cancer, social media, Twitter

## Abstract

**Background:**

As social media becomes increasingly popular online venues for engaging in communication about public health issues, it is important to understand how users promote knowledge and awareness about specific topics.

**Objective:**

The aim of this study is to examine the frequency of discussion and differences by race and ethnicity of cancer-related topics among unique users via Twitter.

**Methods:**

Tweets were collected from April 1, 2014 through January 21, 2015 using the Twitter public streaming Application Programming Interface (API) to collect 1% of public tweets. Twitter users were classified into racial and ethnic groups using a new text mining approach applied to English-only tweets. Each ethnic group was then analyzed for frequency in cancer-related terms within user timelines, investigated for changes over time and across groups, and measured for statistical significance.

**Results:**

Observable usage patterns of the terms "cancer", "breast cancer", "prostate cancer", and "lung cancer" between Caucasian and African American groups were evident across the study period. We observed some variation in the frequency of term usage during months known to be labeled as cancer awareness months, particularly September, October, and November. Interestingly, we found that of the terms studied, "colorectal cancer" received the least Twitter attention.

**Conclusions:**

The findings of the study provide evidence that social media can serve as a very powerful and important tool in implementing and disseminating critical prevention, screening, and treatment messages to the community in real-time. The study also introduced and tested a new methodology of identifying race and ethnicity among users of the social media. Study findings highlight the potential benefits of social media as a tool in reducing racial and ethnic disparities.

## Introduction

Cancer is a major public health problem, impacting more than 14 million men and women in the United States. As of January 2014, an estimated 1.6 million additional new cancer cases will be diagnosed among Americans in 2015 [1]. African Americans have experienced higher age-adjusted mortality rates when compared with Caucasians [1,2]. Many factors contribute to these disparities. Socioeconomic status (SES) as a whole, along with its primary components, including education, income, employment status, and neighborhood appear to be obvious correlates of cancer mortality disparities [3-5]; however, other factors that are not clearly understood may also play a role [2,6,7]. One important factor that could particularly contribute to improved cancer prevention and thereby possibly reduce cancer disparities is knowledge and awareness about cancer.

Knowledge and awareness about the four cancers with the highest incidence and mortality among adults in the United States, namely lung, breast, prostate, and colorectal cancer, has been shown to differ by race and ethnicity [8-17]. Lung cancer is a good example of these differences. It is widely known that cancer of the lung is the leading cause of cancer death in the United States among both men and women and that tobacco smoking is the most significant and preventable cause of the disease. However, findings from one study [11] suggested that two-thirds of US women could not correctly identify lung cancer as the leading cause of cancer death, and this lack of knowledge was greatest among African American women [11]. In terms of breast cancer, evidence has shown that breast cancer knowledge also greatly varies by racial and ethnic group. One study [13] showed that African American women were generally unaware of disparities in breast cancer mortality. Furthermore, one study found that South Asian women tend to have better knowledge of age-related breast cancer risks when compared with black and white women [14]. Knowledge and awareness about both prostate and colorectal cancers have been shown to be low among US adults overall and particularly among low SES groups [12,15-17]. These examples highlight the importance of promoting knowledge about cancer among some segments of the US population, particularly among groups with the highest cancer burden.

Today, social media outlets including Twitter, Facebook, and Instagram, are popular online platforms to engage in communication about any and everything, and many studies [18-32] have begun examining the importance of social media in reaching larger audiences for promotion of public health knowledge and patient advocacy. Twitter has become a very popular site and application for the exchange of health-related information. Twitter allows users (individual users and organizations) to exchange information with other users around the world in real-time, through short messages called "tweets" (less than or equal to 140 characters) posted on a given users’ timeline (ie, the chronologically ordered collection of tweets posted by a given user). Twitter also allows users to re-tweet (repost) other users’ tweets, which promote the exchange of messages to a very large number of individuals. Many health care agencies and public health organizations (ie, local and national organizations and private companies) [21,23,27,33,34] use Twitter as a major online platform for health education and promotion because the majority of Twitter content is publicly available and may provide a novel source of health-related information. In fact, recent studies [35,36] have touted the numerous epidemiological advantages of coupling machine learning techniques with social media mining. Marathe et al [36] discuss the real-time possibilities of understanding disease outbreaks using social media data. Dredze et al [35] state that geo-specific data coupled with the public forum nature of social media (which encourages the sharing of detailed information) creates new public health capabilities not previously seen. Simultaneously, advances in demographic extraction techniques and computational linguistics have allowed for a deeper understanding of user demographics [37,38]. In these studies, Beretta and Burger connected age and gender to linguistic patterns (often word usage). In the case of Beretta [37], user profile images manually labeled by human experts helped to verify the experimental results. Much of the demographic extraction studies have built upon studies originating in the field of psychology, connecting linguistic patterns to demographic elements of participants [39,40]. In Colley’s work [39], participants’ inboxes were examined for linguistic differences differentiating the genders.

In this study, we aim to explore differences in cancer-related tweeting by race and ethnicity, basing our work on Rickford’s assertion of unique vernacular patterns amongst African-Americans [40]. Findings from this study will ultimately contribute to the development and implementation of cost-effective, prevention, and dissemination strategies, delivered through social media messaging, targeting specific subgroups that would benefit from increased cancer knowledge and awareness.

## Methods

### Preprocessing

**Figure 1:**
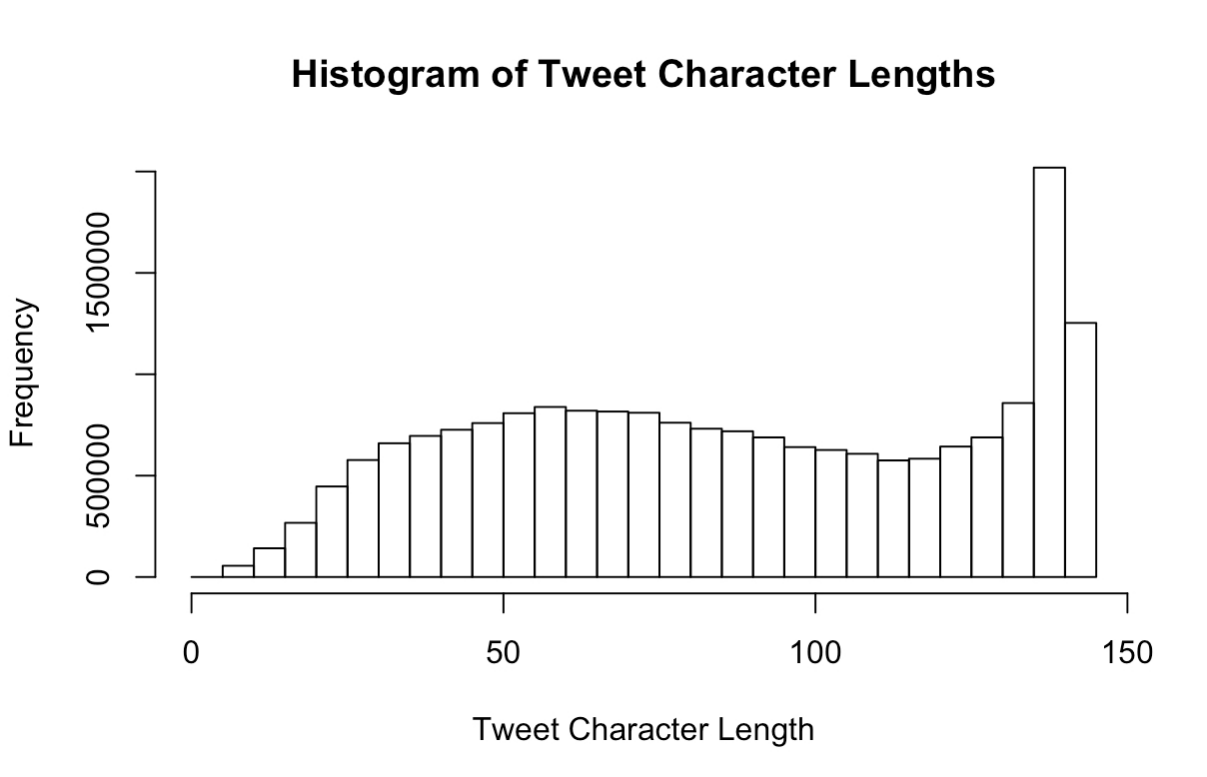
Histogram showing the distribution of Tweet character lengths.

**Figure 2:**
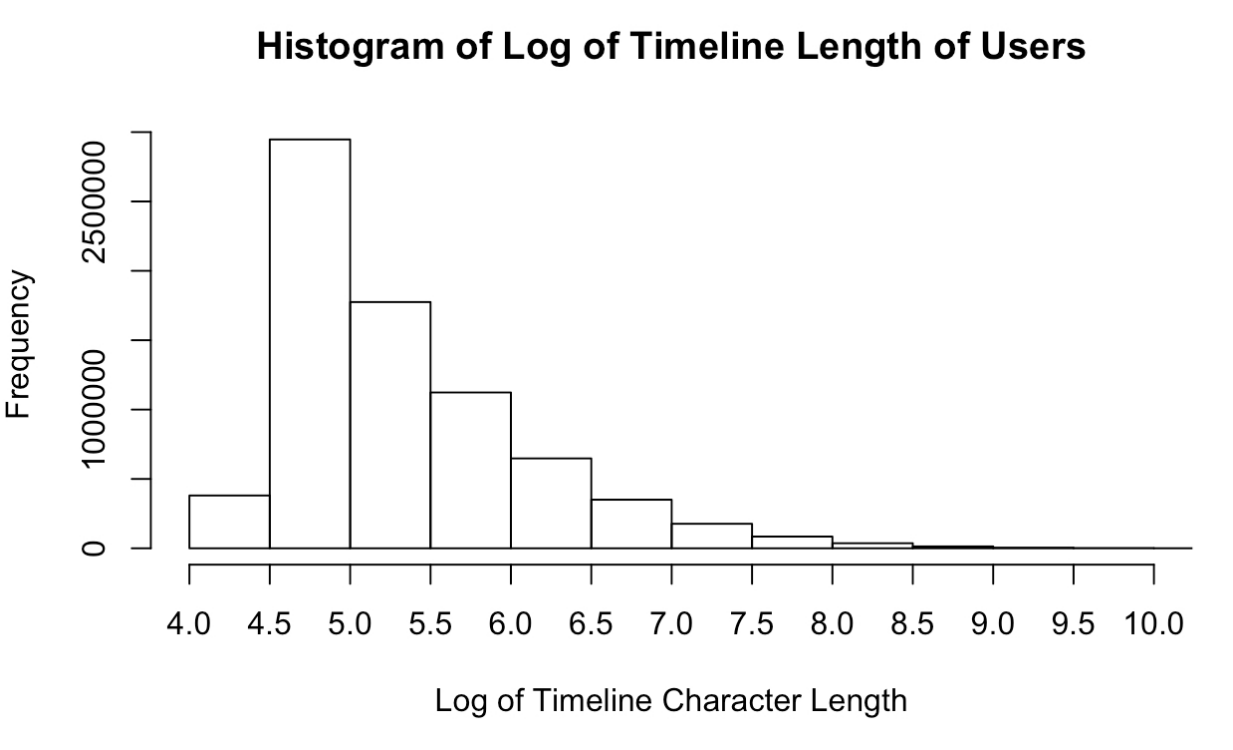
Histogram of the log of the character length of user timelines. We present this graph in log-form due to the wider distribution of character lengths in timelines.

Tweets were collected from April 1, 2014 through January 21, 2015 using the Twitter public streaming Application Programming Interface (API) to collect 1% of public tweets, yielding 281,276,343 tweets. For this study, we restricted our collection to English-only tweets. We provided no restriction on Global Positioning System (GPS) values for each Tweet due to the sparsely available GPS data and instead focus our Tweet location to US-only accounts using an approach introduced later in this paper. Due to a technical issue with our collection system, tweets from May 13, 2014 through July 24, 2014 were not retained. During the data collection period, the Twitter-provided unique user identification (ID) number, tweet, data/time, profile-identified location, and GPS latitude and longitude values were collected (when available). Following the collection of tweets, user timelines were re-constructed by grouping tweets using the unique user ID number. The distribution of character lengths for tweets in the collection are shown in Figures 1 and 2.

The preprocessing procedure for cleaning tweets followed a consistent approach across all collected timelines. Given that the focus was on the predictive power of text, tweets containing linking information outside of the self-contained tweet, predominately non-language elements (ie, URLs, usernames, and re-tweet information) were systematically removed. For example a tweet containing elements such as, "www.t.co", "cnn.com", "@username", and "RT @username" would be removed from the collection. While re-tweeted text may provide information about individuals and/or organizations a user interacts with via Twitter at this scale, we were unable to include all re-tweets using the provided Twitter API due to rate limitations (ie restrictions imposed by Twitter limiting the number of searches we could conduct in a 15-minute period). User timelines (tweets aggregated by user) that contained little information were removed by systematically eliminating those that were shorter than 85 characters from the study. To select this character threshold, we randomly selected timelines of varying length and observed that timelines shorter than 85 characters generally contained fewer than fifteen words, which provided little information to make accurate classifications. These preprocessing methods left us with a final Tweet count of 19,818,236 belonging to 779,653 unique users’ timelines for analysis.

### Identification of Race and Ethnicity

The approach to classifying users’ ethnicities presented in this paper relies on a supervised learning classification approach [41], which requires accurate training data to inform the classification model and also a reliable set of testing data for assessing the accuracy of classifications. To acquire training data indicating the ethnicity of Twitter users, we looked for specific declarative statements within each user’s timeline (ie, statements where users explicitly defined an element of their personal identity). Timelines that contained such declarative statements were labeled accordingly, receiving one of four enumerated keys. These keys indicated the types of ethnicity explored by this study, taking the values of: *Caucasian*, *African-American, Asian,* and *Hispanic.* Examples of declarative statements include: "I am African-American", "I’m Asian", or "I’m a black man." These statements were chosen by manually observing statements around ethnic terms (eg, white, black, Caucasian, African-American, Asian, etc), which determined that many self-identifying statements took on similar forms compared to the declarative statement examples provided above. Although we are aware of the differences in race and ethnicity, this study does not make distinctions between the two types of declarative statements since the end Twitter users who contributed to such statements are not always sound or consistent.

### Classification of Race and Ethnicity

Individual tweets are short, often uninformative messages providing little classification potential for identification of user profile information. This led us to examine users’ timelines, rather than individual tweets, to enhance the accuracy of our classification approach by extracting features consisting of deeper information around users’ activities. Users’ tweets were collapsed into timelines containing the chronological order of their submitted tweets for the 10-month data collection period. This provided a larger text source for identifying descriptive elements indicative of a given user’s ethnicity.

Baseline classification models described in previous work [38] adopt document-term matrices for representing the frequency with which terms appear in a given timeline. Classification algorithms are used to detect vocabulary usage patterns among a common set of users. In this study, a common set consisted of users with the same *self-identified* ethnic background (eg, "I’m African-American" appearing within their timeline). The vocabulary usage patterns detected among the self-identified users were then applied to users who chose not to explicitly define their ethnicity. Two opposing scenarios were examined in this study (1) how timeline synonym expansion can enhance predictive ability; and (2) how dimensionality reduction can enhance predictive ability of users’ ethnicities. These scenarios were born from two ideas. The first is that users often express similar thoughts on social media with varying lexical choices, and secondly, sparsely populated timelines potentially compromising the accuracy of our classifications.

When building the baseline classifier [42-44], based exclusively on users’ choices of vocabulary, we discovered that there was often difficulty connecting some classifiers to specific ethnicities. For example, one ethnic group may often use terms such as *wife, spouse,* and *marriage,* consistently appearing as some of the most identifying terms for that group*.* Having identified that Twitter users often used varying terms to describe the same concept, we expanded tweets with additional vocabulary in an attempt to increase lexical overlapping of group member term usage to easily segment profile types. Using part-of-speech tagging, we identified nouns and verbs within tweets. Then for each tweet, using Wordnet (a lexical database where nouns, verbs and adjectives are collected into sets of cognitive synonyms) [45,46], the top five synonyms, when available, for each noun and verb were appended to the tweets, resulting in expanded tweets while retaining their original meanings. This allowed for more frequent overlap between tweet term usage among racial and ethnic groups and a more accurate classification algorithm. To the best of our knowledge, using synonym expansion of tweets to enhance the bag-of-words feature set has not been explored in detecting the ethnicity of Twitter users.

Latent Dirichlet allocation (LDA) [47] is a statistical method for computing abstract topics of a given document using the co-occurrences of terms within the documents of a corpus. Our second ethnic classification approach used LDA to detect patterns among topics rather than vocabulary usage by first converting tweets into topics. We acknowledge that LDA is typically used for topic detection in long documents and its limitation when applied to topic detection from short text. Nevertheless, by our study design, all tweet text contributed by a Twitter user were first aggregated to generate the user’s total writing record on Twitter, after which LDA was applied onto the aggregated writing record of a user (averaging 324 characters). In Figures 1 and 2, we summarize tweet and total tweet writing record (user timeline) length of the collection of tweets examined herein. This author-based aggregation step greatly mitigates the sparsity issue of short input text to the LDA model. It is noted that the above preprocessing step is also popularly adopted when topic modeling is applied to Twitter data [48-50]. Using LDA topic distributions to represent timelines resulted in a reduction of features (variables used for classifying the ethnicity of a user-for example, these variables consisted of frequency counts of stemmed-words such as "togeth", "damnnn", and "sharp", which generally indicated an African American user, and "newyork", "lifetime", and "whatchya", which were strongly associated with Caucasian users) by 99.7% while improving classification accuracy for some ethnic groups. The number of abstract topics, and thus the number of features representing Twitter timelines, was decided on systematically by iteratively building classification algorithms with increasing larger topic sizes. Accuracy of the model within this corpus of timelines peaked at approximately 45 abstract topics, which was then adopted for each testing set. In this approach, we aimed to reduce the number of features representing the activities of each Twitter user. Having reduced users’ timelines to representation comprised of LDA topic distributions, we then adopted a Support Vector Machine classification approach with a radial basis function kernel for our classification algorithm. This method was chosen for its demonstrated ability to perform well with text data and is consistently considered the best approach in text classification studies [51].

**Figure 3:**
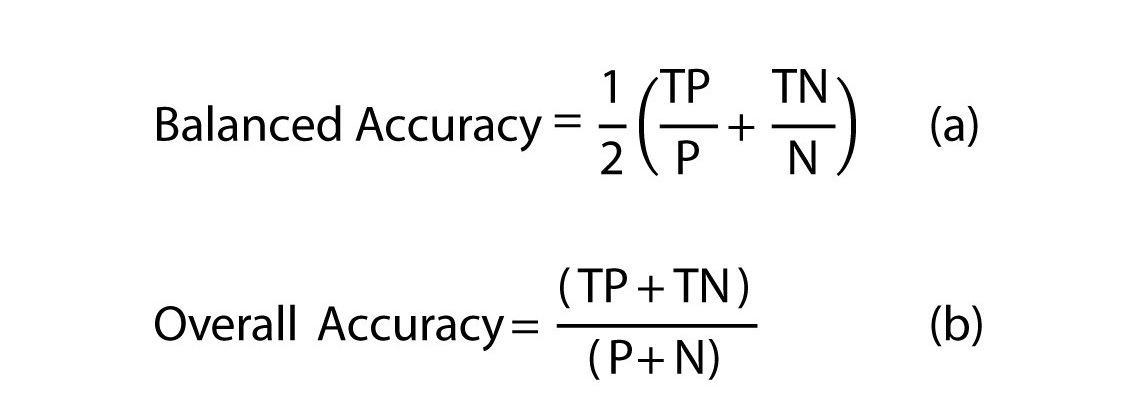
Balance and overall accuracy equestions.

We used ten-fold cross validation to test the accuracy of the models. The labeled dataset was divided into ten, equally sized bins. Nine of the ten bins were used to train the model, while the remaining bin was used for testing. We iterated over the bins ten times, reserving a new bin for testing with each additional iteration. Due to the unbalanced nature of our dataset, we chose two evaluations metrics. First, for each ethnicity, we computed the balanced accuracy (Equation a, Figure 3), a performance metric intended for unbalanced classes [52]. Second, we provided the overall accuracy for all ethnicities (Equation b, Figure 3), as well as the accuracy for Caucasians and African Americans (the two groups focused on in the second part of this study). In addition, we provided a confusion matrix of the classification results (results for text classification with synonym expansion and results for the topic-based method) to give further details of the classification performance.

### Statistical Analysis

All statistical analysis for this study was carried out using R Statistical Software Package. To measure the statistical significance of the observed differences between groups, *t* tests were conducted with pairwise comparisons of ethnic groups (ie, Caucasian vs African American, Caucasian vs Hispanic, etc). We tested the hypothesis that there were no statistically significant pairwise racial and/or ethnic group differences in cancer term usage during each month of the study period. Because pairs of ethnic groups were tested independently of one another, no adjustments for multiple comparisons were made. *P* values <0.05 were considered statistically significant.

## Results

To evaluate the success in the classification of race and ethnicity, we compared the accuracy of text classification with synonym expansion against the topic-based method (Tables 1 and 2). We found that the accuracy of text classification with synonym expansion outperformed the topic-based approach in most cases. Using the synonym expansion approach, we achieved the following accuracies for correctly identifying user ethnicities: 88.87% among Caucasian users, 81.26% among African-American users, 72.32% among Asian users, and 69.07% among Hispanic users. The overall accuracy for all groups using this approach was 76.07%. Using topic detection, we observed no improvement in overall accuracy at 55.59%. Among the groups we also observed a lower accuracy score (Caucasian, African-American, Asian, and Hispanics resulting in 71.89%, 68.32%, 53.43%, and 54.50% respectively). We suspect topic detection classification produced lower accuracy scores due to the loss of nuanced lexical differences between ethnic groups lost during the feature reduction process.

**Table 1:** Text classification with synonym expansion model classification and accuracy results.

Race and ethnicity		%
Balanced accuracy		
	Caucasian	88.87
	African American	81.26
	Asian	72.32
	Hispanic	69.07
Overall accuracy		
	All groups	76.07
	Caucasian and African Americans	88.30
		

**Table 2:** Confusion matrix.

Classification	Reference, n			
	Caucasian	African American	Asian	Hispanic
Caucasian	1067	117	49	71
African American	890	1286	337	380
Asian	26	10	39	35
Hispanic	7	7	25	54

Given the higher overall accuracy, as well as the high accuracies among Caucasian and African-American users, we selected the synonym expansion approach for classifying the remaining unlabeled users within the collection. Additionally, we elected to exclude users classified as Asian and Hispanic from this study for multiple reasons. First, the population sizes where users declared ethnicities of these types were markedly smaller than populations of Caucasians and African-Americans. In addition, we believe we may have excluded some Asian and Hispanic users by limiting the Tweet collection to English-only tweets. The combination of these complications (small population sizes and the restriction of English-only tweets) is likely reasons for the reduction in accuracy among these groups and their subsequent exclusion from the study.

In this study, we have established and tested a systematic method for detecting ethnicities among Twitter users. Using the more accurate approach, text classification with synonym expansion, we detected and assigned ethnicities to all users within the collection consisting of 19,818,236 tweets posted by 779,653 unique users. Tweets were divided by posting date into nine months, accounting for the ten-month study period with portions of May and July and the entirety of June lost due to system failure. Various descriptive statistics were calculated to describe the health effects extracted from the dataset.

As shown in Table 3, the number of unique users varied widely by race and ethnicity. To detect significant differences in term usage between ethnic groups, each term contribution was normalized by the percentage distribution of population. Additionally, the term frequency for each ethnic group is provided without normalization. The number of unique users from each ethnic group was examined for each month. Caucasian users dominated the dataset (92.32%, 719,798/779,653), while African-American users often represented 7.12% (55,549/779,653) of the population, and both Asian and Hispanic users made up a small percentage of the overall population (0.55%, 4306/779,653). We were less confident in predications of Asian and Hispanic ethnicity among users based on the smaller training set as well as the lower accuracy values among these ethnic groups.

**Table 3:** Distribution of unique active Twitter users during each month of the study period by race and ethnicity.

Month	Race and ethnicity, n (%)				Total
	African American	Caucasian	Asian	Hispanic	
April	49,104 (9.72)	452,924 (89.64)	1289 (0.25)	1935 (0.38)	505,252
May^a^	40,956 (12.76)	277,169 (86.36)	1177 (0.37)	1646 (0.51)	320,948
July^a^	43,349 (9.58)	405,185 (89.57)	1661 (0.37)	2191 (0.48)	452,386
August	54740 (7.91)	632,687 (91.47)	1820 (0.26)	2466 (0.36)	691713
September	52,224 (10.16)	457,300 (89.02)	1789 (0.35)	2417 (0.47)	513,730
October	50,120 (11.07)	398,440 (88.02)	1763 (0.39)	2371 (0.52)	452,694
November	50,060 (10.80)	409,125 (88.30)	1762 (0.38)	2370 (0.51)	463,317
December	48,247 (11.20)	378,412 (87.86)	1727 (0.40)	2292 (0.53)	430,678
January	30,707 (15.62)	162,682 (82.75)	1435 (0.73)	1780 (0.91)	196,604

^a^Tweets from May 13, 2014 through July 24, 2014 were not retained due to a system outage.

This study focused on the social media attention given to site-specific cancers and differences by race and ethnicity. Specifically, Twitter timelines were examined for the frequency of occurrence of the following terms: "cancer", "breast cancer", "prostate cancer", "colorectal cancer", and "lung cancer." These terms were detected using methods adopted in previous studies examining discussions about specific health topics on Twitter [53]. We are aware of other work [54] that distinguishes between medically-related use of the term "cancer" and non-medically related uses. However, when examining our own dataset, by sampling 200 randomly chosen tweets, we observed only 8.5% (17/200) of tweets were used in the context of Zodiac signs and 2.0% (4/200) referred to destructive practices (eg, "he was a cancer to the community"). We suspect the low percentage of non-medically related usage may be a result of the cleaning process performed, where tweets containing URLs were stripped from the collection (ie, horoscope tweets often contain links to an extended version of the horoscope). Furthermore, we examined samples of each of the bi-gram terms of interest (eg, "breast cancer", "prostate cancer", "colorectal cancer" and "lung cancer"). We observed no uses of the term "cancer" in a context other than the medical terminology when examining these samples, presumably because of their specificity. We retained the uni-gram term in our study for comparison; however, we focus the discussion on the results related to the bi-gram terms.

**Figure 4:**
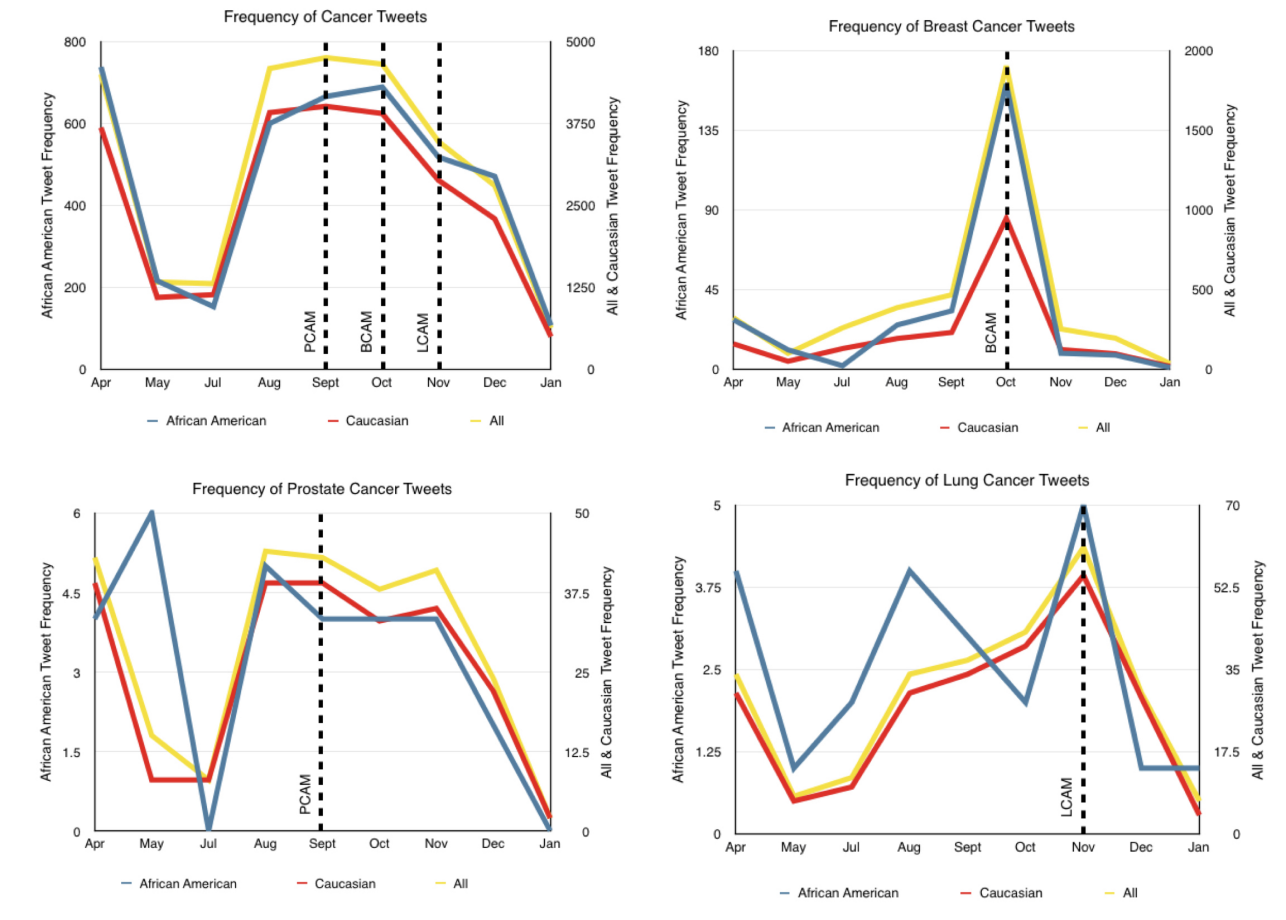
Monthly frequency of cancer terms by race/ethnicity (African American, left axis; Caucasian, right axis), and all Twitter users (right axis). Cancer terms are "Cancer" (top left), "Breat Cancer" (top right), "Prostate Cancer" (bottom left), and "Lung Cancer" (bottom right). It is important to note the sharp decreases seen following cancer awareness months (Prostate Cancer Awareness Month [PCAM, September], Breast Cancer Awareness Month [BCAM, October], and Lung Cancer Awareness Month [LCAM, November]), particularly among African Americans. Both groups are seen returning to lower frequencies following awareness months; however, this observation is more prevalent among African Americans, specifically following BCAM.

First, we examined user activity by ethnicity during each month of the study period to understand seasonal peaks in term usage on Twitter (Table 3). We then counted the frequency of cancer terms for each month and by ethnicity. The types of cancer examined in this study include: breast cancer, prostate cancer, colorectal cancer, and lung cancer. For "cancer"-related tweets, we counted the detection of the following keywords: *benign, cancer(s), cancerous, carcinogen, carcinogenic, chemo, chemotherapy, chemotherapeutic, cyst(s), growths, leukemia, lymphoma, malignant, metastases, metastasis, metastatic, neoplasm, neoplasm, oncologist, oncology, radiation, radiotherapy, recurrence, and tumor(s)*. These set of terms were adopted from a previous study [55]. For specific cancer types, we used the National Institute of Health’s website for other disease synonyms. For breast cancer, we searched for: *breast cancer, breast carcinoma, cancer of the breast, malignant neoplasm of (the) breast, malignant tumor of (the) breast, and mammary cancer.* For colorectal cancer, we searched for: *colorectal cancer and colon cancer.* For lung cancer, we searched for: *lung cancer, cancer of bronchus, cancer of the lung, lung malignancies, lung malignant tumors, lung neoplasms, malignant lung tumor, malignant neoplasm of lung, malignant tumor of lung, pulmonary cancer, pulmonary carcinoma, pulmonary neoplasms, and respiratory carcinoma.* Finally, for prostate cancer, we searched for: *prostate cancer, cancer of the prostate, malignant neoplasm of the prostate, prostate carcinoma, prostate neoplasm, prostatic cancer, prostatic carcinoma, and prostatic neoplasm*. All searches were conducted within our tweet collection. Observable differences between Caucasian and African American groups were present in almost all of the chosen cancer terms across each month of the study period (Figure 4). However, observations of certain terms, namely "colorectal cancer", showed prominently lower frequency counts when compared with other terms and thus were not shown graphically.

Finally, we examined the differences in term usage by race and ethnicity within each month of the study period using *t* tests of pairwise differences (Table 4). During most months, the Caucasian and African American groups showed statistically significant differences in terms of Twitter activity. However, in terms of colorectal cancer, we observed few months where there was a statistically significant difference between these two groups. Again, we suspect this is a result of the limited number of users discussing this particular type of cancer via Twitter. Lastly, lung cancer showed a statistically significant difference between Caucasians and African Americans during the months of September through December, excluding other months.

**Table 4:** Statistical significance of pairwise differences in cancer term usage between African Americans and Caucasians during each month of the study period^a^.

Month	Cancer term, *t* test				
	"Cancer"	"Breast cancer"	"Prostate cancer"	"Colorectal cancer"	"Lung cancer"
April	0.00003	0.053025	0.014894	0.025347	0.080356
May	0.008194	0.584394	0.122251	0.095581	0.510364
July	0.013599	<0.0001	0.006656	0.157299	0.890133
August	<0.0001	0.001168	0.157209	0.312076	0.165111
September	<0.0001	0.00007	0.017132	0.157299	0.013196
October	<0.0001	<0.0001	0.242175	0.974206	0.000162
November	<0.0001	<0.0001	0.027708	0.014306	0.000631
December	0.000266	0.000001	0.027575	0.317311	0.000067
January	0.241671	0.00945	0.1573	0.083265	0.91944

^a^Each user’s total term usage was calculated by summing the frequency with which cancer terms appeared in their timeline.

## Discussion

### Principal Findings

In this study, we observed interesting patterns of media attention given to specific cancer terms among unique Twitter users during a 9-month period in 2014. With a focus on cancer in general, and breast, prostate, and lung cancers specifically, which are the leading cancers among men and women in the United States, we observed some variation in the frequency of term usage during and after specific months known to be cancer awareness months, specifically September (Prostate Cancer Awareness Month [PCAM]), October (Breast Cancer Awareness Month [BCAM], and November (Lung Cancer Awareness Month [LCAM]). Interestingly, colorectal cancer, the third most common cancer in both men and women [1], received the least attention on Twitter among users sampled in this study across the board. We observed differences in frequency of use of each of the cancer terms of interest throughout the duration of the study period by race and ethnicity which we hypothesize are related to observable cancer disparities in the United States. These findings highlight the necessity for increased cancer awareness in the population and the importance of studying how individuals use social media to spread information about cancer, which could ultimately be utilized in the future for real-time cancer awareness intervention implemented through Twitter (and other social media channels).

Overall, we found that the frequencies of mentions of "cancer" among Caucasian and African American users were similar in terms of seasonal increases or decreases, although it appeared that African Americans maintained a higher percentage of normalized Tweet frequency of this broad term compared to the Caucasian group. In terms of the frequencies of mentions of "breast cancer", Caucasian users consistently had a higher percentage of use during all months of the study period. As expected, the frequency of use of this term was highest during BCAM, with a dramatic decrease in the months following, ultimately returning to levels lower than observed leading up to BCAM. This was true among both Caucasians and African Americans; however, there was a steeper decline in the mentions of "breast cancer" on Twitter among African Americans following BCAM.

This may be an area that can be the focus of future interventions aimed at increasing breast cancer awareness throughout the year, which could contribute to increased knowledge, improved within-guidelines screening rates, and increased preventive activities among groups with a disproportionate disease burden. For example, weekly Twitter chats hosted by the #bcsm ("breast cancer social media") community have been shown to raise awareness and decrease medical anxiety in patients [31]. Identifying individuals who were active during BCAM and inviting them to participate in Twitter chats could be a way to build an engaged, on-going community of active participants in discussions about cancer in groups with a disproportionate disease burden. Chats can be facilitated with the use of a consistent hashtag, which is a convention on Twitter designed for marking tweets about specific topics. Enlisting experts and celebrities to guest host chat sessions may be a way to promote sustained engagement, particularly because people tend to prefer health-related messages on social media that come from sources with high status and credibility [25]. These interventions would leverage Twitter’s capabilities to deliver just-in-time information and social support, involving individuals proactively in evidence-based discussions about cancer throughout the year [56]. This intervention method may be appropriate for other types of cancer as well.

During PCAM, there was a substantially higher frequency of discussion of prostate cancer among Caucasians compared to African Americans. In July and January, among Caucasian users, we observed the lowest levels of prostate cancer discussion. Conversely, among African Americans, we observed a steady decrease in prostate cancer discussion from August through January. Following PCAM, we observed a decline in the frequency of use of the term "prostate cancer" among both groups; however, these declines were slower than that observed with other cancer awareness campaigns. For example, when examining the frequency of use of the term "lung cancer", we observed a peak in November (LCAM) and then a dramatic decrease to levels lower than observed in the months prior to LCAM.

The months following cancer awareness month campaigns also presented interesting findings. While awareness month campaigns (eg, PCAM, BCAM, LCAM) could be considered successful in promoting discussion around various cancer topics, our findings suggest that these campaigns as evidenced by mentions of cancer terms via Twitter during specific cancer awareness months, did not appear to sustain long-term interest and discussion. This phenomenon was particularly evident when examining breast cancer discussion frequency, but was also present in both lung cancer and prostate cancer social media activity. In fact, our findings showed that racial and ethnic groups often returned to a state of lower participation following awareness campaigns when compared with preceding months. Notably, this reduction in discussion frequency appeared to be more prevalent among minority groups. For example, African Americans reduced their participation by 73% in the month following BCAM when compared with months preceding the program. Among Caucasians, we also saw a drop in participation where we observed only a 47% reduction. Similarly for LCAM, we observed a 50% drop among African Americans compared with a 25% drop in the Caucasian cohort. Finally, in terms of discussion of colorectal cancer, we saw poor participation throughout the months of the study. This could be an indication of poor marketing or the taboo nature of the topic among some populations as well as lack of collection of tweets during Colorectal Cancer Awareness Month (CRCAM) due to a technical issue with our data collection system.

These drops in participation are likely related to media exposure and framing, two media effects that are mediated by structural determinants of health (eg, SES, race, ethnicity) [57]. Media exposure is the extent to which individuals encounter information about cancer in the mass media rather than specifically seeking it out; framing describes how topics like cancer are discussed in the mass media. This finding points to the need for interventions that use appropriate framing for minority populations. For example, using Twitter to share narratives about cancer could be particularly fruitful. Digital narratives have been successfully implemented in interventions aimed at raising awareness and improving screening rates in breast cancer, colorectal cancer, and prostate cancer [57-59]. Although tweets are short, they could be used to share short-form narratives or could be employed in conjunction with other storytelling techniques to provide engaging narratives about cancer with the aim of raising awareness and disseminating credible information about cancer to populations with a disproportionate disease burden [60].

With the growing popularity of social media and the previously unavailable personal insights it offers, social media mining presents new opportunities and methods applicable to epidemiologic research. Existing studies have examined the health impacts of social media, as shown in previous work [32] where researchers concluded that Tobacco Control Programs are ineffective in capitalizing on social media platform’s potential. In addition, Thackeray et al examined the frequency of breast cancer-related tweets during BCAM [25] and concluded that Twitter could be a tool used for increasing health conversations to maximize health marketing. In the present study, we examined how new text mining techniques can be used to extract a user’s race and ethnicity through lexical analysis, thereby providing a new opportunity to inform future studies to potentially address racial and ethnic health disparities. However, this work can be further expanded to examine differences across other demographic characteristics, as well as the investigation of disparities with respect to diseases other than cancer. Finally, understanding a social media user’s demographic makeup also presents new opportunities for appropriately targeting health education materials.

### Limitation

There were limitations of this study that should be considered. First, our findings provide only a glimpse of all tweets, focused on cancer-specific topics among users without private Twitter accounts during one year. Thus, there could very well be an underestimation of the frequency of cancer-focused discussion via Twitter. Relatedly, it is possible that tweets of interest were missed due to our choice of keywords or use of alternate terms and/or spellings of some words among the users. It is possible that we missed tweets of interest based on the keywords we have chosen to examine and, consequently, the true frequencies of cancer-related tweets may be higher than what we currently examined in the analysis. Nevertheless, our large-scale systematic examination of 779,653 unique Twitter users and their tweets contributed during a 9-month period still provides a meaningful glimpse into users’ social media activity related to general or specific cancer topics. Due to the scope and length limit of this manuscript, we choose to report several representative case studies using the most popular cancer terms concerned by Twitter users. As demonstrated through these multiple case studies, commonly enabled by the proposed approach, the new method has the promise to be generically applicable for detecting, tracking, and comparing user interests regarding other cancer or disease topics. In addition, due to technical issues with our collection system, we were unable to retain collected tweets from the middle of May through the end of July 2014, which could have contributed to the very low frequency of use of the term "colorectal cancer". In addition, March, which is CRCAM, was not included in our collection period and could also contribute to the low frequency of the term "colorectal cancer." Another possibility is that not all public tweets were delivered from the Twitter public API; but there is no way to determine the likelihood of this possibility. The collection period excluding winter and post-holiday months (late January to March) could potentially miss important patterns that may emerge through the analysis of this time period.

And finally, because several regional, temporal, and country-specific factors may have some influence on the contents of information shared or communicated via Twitter, we went to considerable lengths to limit our dataset to US-based users. Ideally, we would have liked to filter our dataset by a Twitter-provided variable, distinguishing US-based users from non-US-based users. However, because Twitter does not provide this information, we chose to adopt an alternate method for the extraction of US users by looking at the "Location" portion of a user’s profile. This is a free-text area provided by Twitter where users can input information such as New York or San Francisco, California, excluding users with non-US locations in their profile. This method was chosen for the following two reasons: (1) only a small fraction of users provide geo-tagged tweets, and (2) it is difficult to assume that geo-tagged tweets taken internationally do not belong to a US-national. Geo-tagging of tweets varies in location for a given user and, therefore, does not provide an accurate understanding of the location a user defines as home.

### Conclusion

This study demonstrated that social media can serve as a very powerful and important tool in implementation and dissemination of critical cancer education and awareness messages to the community in real-time. These findings could help improve future social media studies, identify trends within groups of users, and target group-specific health education literature by learning users’ characteristics through language differences. This study also introduced and tested a new methodology for identifying race and ethnicity among users of social media, which presents a unique opportunity to study risk profiles, risk factors and behaviors for several conditions by race and ethnicity and has significant implications in reducing disparities through targeted intervention and dissemination of evidence-based information tailored to specific racial and ethnic groups.

## Abbreviations

API: Application Programming Interface
BCAM: Breast Cancer Awareness Month
CRCAM: Colorectal Cancer Awareness Month
ID: identification
GPS: Global Positioning System
LCAM: Lung Cancer Awareness Month
LDA: Latent Dirichlet allocation
PCAM: Prostate Cancer Awareness Month
SES: Socioeconomic status
